# Physical and chemical data of WS_2_ platelets and thickness-dependent photoresponses

**DOI:** 10.1016/j.dib.2018.08.118

**Published:** 2018-08-30

**Authors:** Thanh Tai Nguyen, Malkeshkumar Patel, Dong-Kyun Ban, Joondong Kim

**Affiliations:** a Department of Electrical Engineering, Incheon National University, 119 Academy Rd. Yeonsu, Incheon 406772, Republic of Korea; b Photoelectric and Energy Device Application Lab (PEDAL), Multidisciplinary Core Institute for Future Energies (MCIFE), Incheon National University, 119 Academy Rd. Yeonsu, Incheon, 406772, Republic of Korea

## Abstract

In this data article, the properties of WS_2_/ZnO type-I heterostructure which corresponds to the research article “Vertically trigonal WS_2_ layer embedded heterostructure for enhanced ultraviolet-visible photodetector” (Nguyen et al., 2018) are presented by characteristics of WS_2_ layer, diode properties, and thickness dependent photoresponses. The device performances under the effect of rapid thermal processing (RTP) is presented. The WS_2_ platelets grown by large area sputtering method (Nguyen et al., 2018) was characterized in term of morphology and chemical elements distribution by using transmission electron microscope (TEM), energy dispersive spectroscopy (EDS) and X-Ray photoelectron spectroscopy (XPS). Diode characterization of WS_2_/ZnO like rectifying ratio, ideal factor and barrier height are presented. The variation of photocurrent of ITO/WS_2_/ZnO/FTO/glass photodetector, its dependence on the WS_2_ thickness and influence of post- thermal treatment are presented.

**Specifications Table**

**Table:** 

Subject area	*Physics, Electrical Engineering*
More specific subject area	*Photodetector*
Type of data	*Figures, Table*
How data was acquired	Transmission electron microscope *(TEM, TALOS F200X)*
	*Energy dispersive spectroscopy (EDS, JEOL, JSM_7001F)*
	*X-ray photoelectron spectroscopy (XPS, PHI 5000 VersaProbe-II, ULVAC)*
	*Potentiostat/galvanostat (PGStat, ZIVE SP1, WonA Tech)*
	*Function generator (MFG-3013A, MCH Instruments)*
Data format	*Analyzed*
Experimental factors	*Current-Voltage (I-V) characteristics: Linear sweep voltammetry, scan range: −0.6 to 0.6 V, positive direction, scan rate _100_mV/s*
	*Mott-Schottky: Potentiodynamic impedance spectroscopy, scan range: − 1 V to 0.2 V, step size: 25 mV, A/C signal amplitude: 10 mV, frequency range: 1 MHz−100 Hz, normal speed.*
	*Transient photoresponse: Chronoamperometry, light source: ultraviolet (λ = 365 nm), applied voltage: -1V, pulsed light frequency: 10 Hz, light intensity: 6 mW cm*^*−2*^.
Experimental features	*WS*_*2*_*/ZnO heterostructure properties, effect of heat treatment and WS*_*2*_*thickness on the photocurrent of device*
Data source location	*Incheon National University, Incheon-406772, South Korea*
Data accessibility	*The data are available with this article*
Related research article	*T.T. Nguyen, M. Patel, D.K. Ban, J. Kim. Vertically trigonal WS*_*2*_*layer embedded heterostructure for enhanced ultraviolet–visible photodetector. J. Alloys Compd. 768, 2018, 143–149**[1]*.

**Value of the Data**

• The data relates to chemical states of WS_2_ platelet could be useful to study the defect engineering of WS_2_ material
• WS_2_/ZnO type-I heterostructure design is efficient for the large scale transitional metal dichalcogenides (TMDs) for optoelectronics.
• Effect of the thickness of WS_2_ layer was investigated for the photocurrent profiles.

## 1 Data

**Fig. 1:**
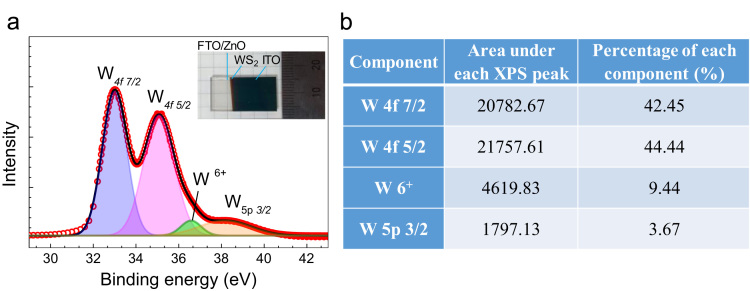
(a) The XPS spectra of tungsten in WS_2_ sample with the inset of image of ITO/WS_2_/ZnO/FTO device. (b) Summary of the quantity of each oxidation state of tungsten in WS_2_ sample.

**Fig. 2:**
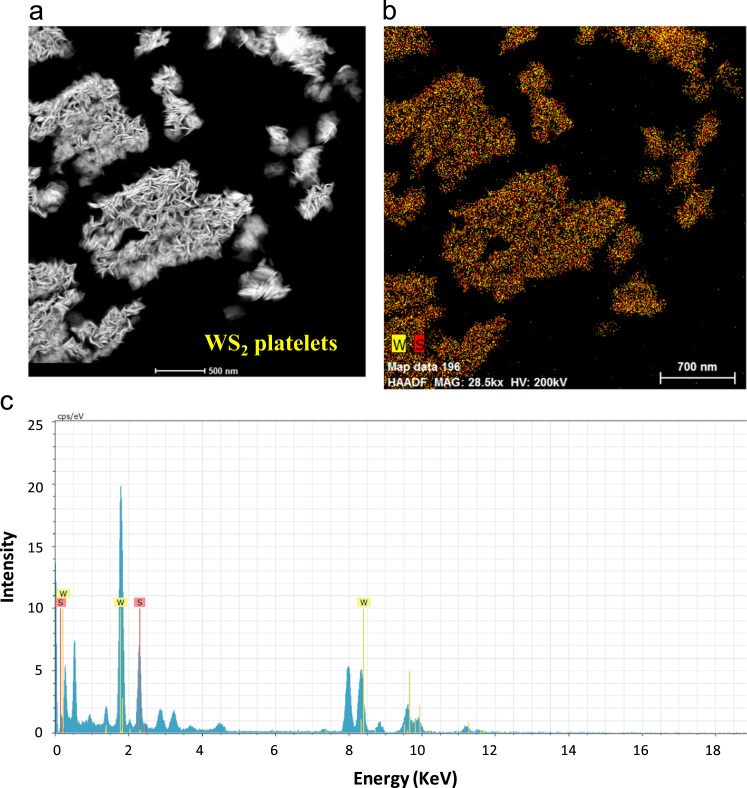
Transmission electron microscope analysis of WS_2_ platelets. (a) Low resolution TEM image and (b) elemental mapping of WS_2_ platelets, (c) energy dispersive spectra of WS_2_ platelets presents the elemental W and S.

**Fig. 3:**
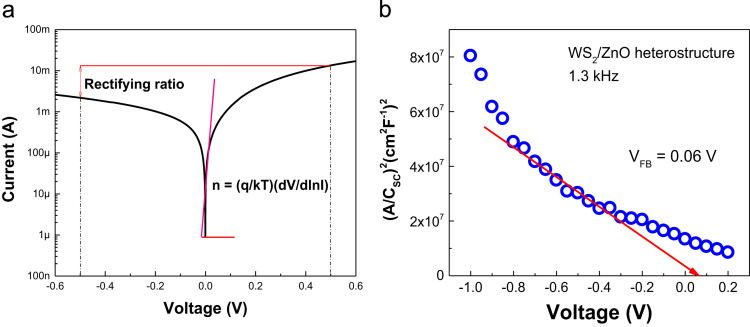
(a) Semi-log current-voltage (I-V) plot and (b) Mott-Schottky characteristic of WS_2_/ZnO heterostructure photodiode. Here n and V_FB_ are the diode ideality factor and flat band potential, respectively.

**Fig. 4:**
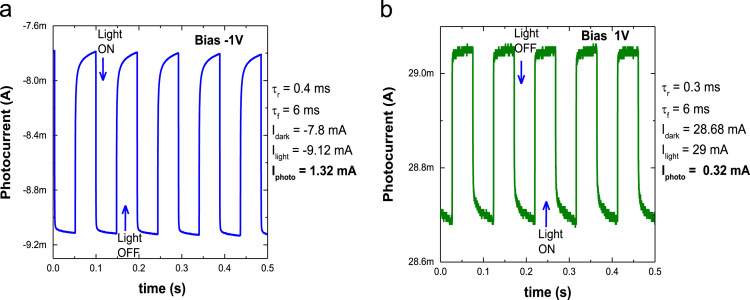
Current – time profile of WS_2_/ZnO photodetector obtained at bias of (a) −1 V and (b) +1 V.

**Fig. 5:**
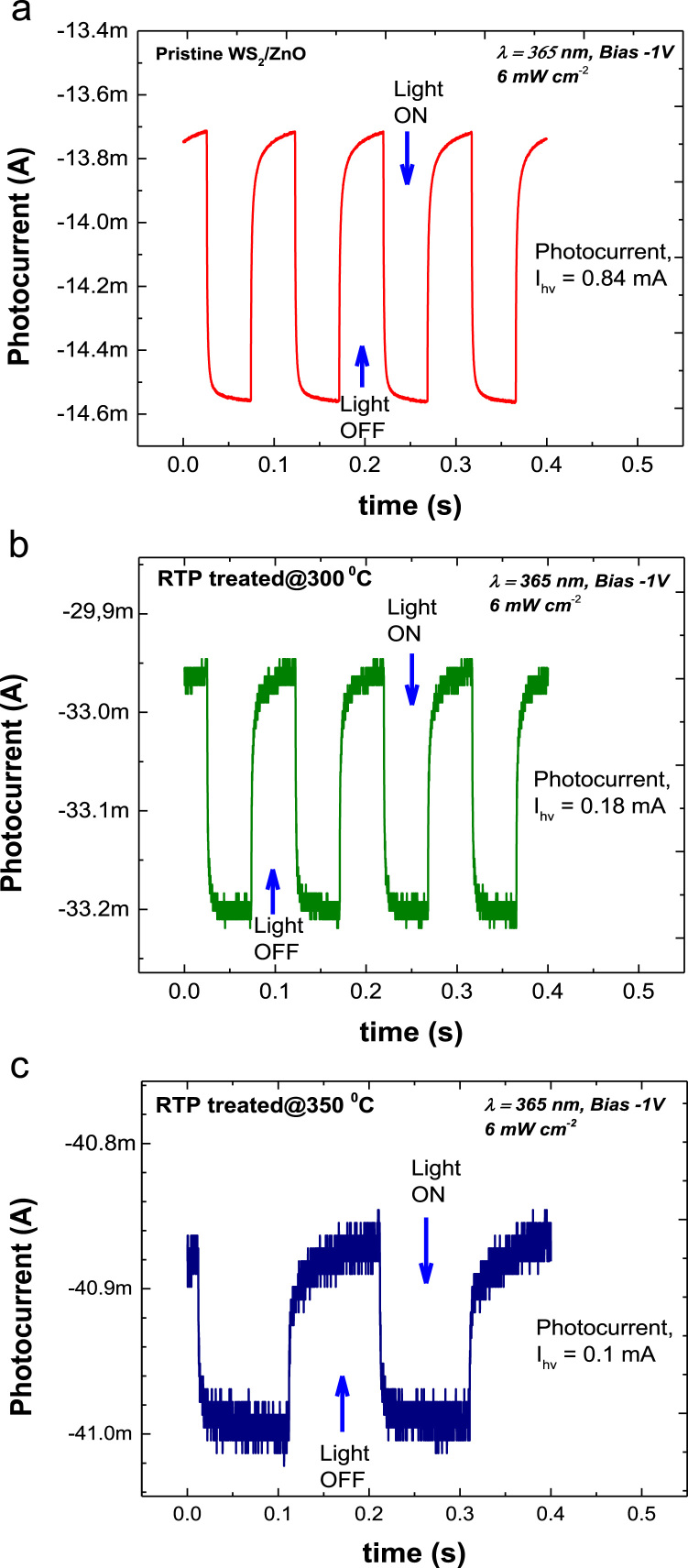
Current-time profiles of WS_2_/ZnO heterostructure obtained under same operation conditions. (a) Pristine, (b) RTP treated at 300 °C and (c) RTP treated at 350 °C. (RTP condition: flowing Argon at 0.5 lpm, pressure of 20 mTorr, holding time of 5 min).

**Fig. 6:**
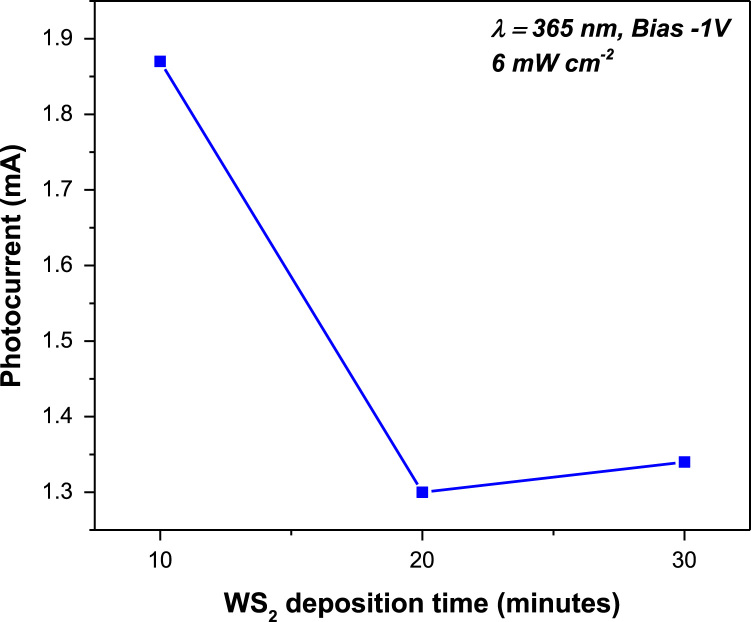
The photocurrent vs WS_2_ deposition time of the ITO/WS_2_/ZnO/FTO device under UV illumination.

The quantities of various oxidation states of tungsten are presented in Fig. 1 by using XPS measurement. Further, Fig. 2 shows the morphology of vertical WS_2_ platelets grown by sputtering method [1] and the distribution of W and S in the WS_2_. The diode properties of WS_2_/ZnO structure includes the rectifying ratio, diode ideality factor and potential barrier height are presented in Fig. 3. In addition, the transient photocurrent profiles of WS_2_/ZnO device are presented in Fig. 4 (at ± 1 bias). The parameters used to simulate the band diagram of the WS_2_/ZnO heterostructure are summarized in the Table 1 for Solar Sell Capacitance Simulator (SCAPS) [1]. Later, considering properties of vertically grown WS_2_ as well as WS_2_/ZnO heterostructure, the effect of post-treatment by RTP treatment and WS_2_ deposition time on ITO/WS_2_/ZnO/FTO device performance is shown in Figs. 5 and 6, respectively.

**Table 1:** Parameters in SCAPS simulation.

**Material**	**Thickness (nm)**	**Bandgap (eV)**	**Doping concentration (cm**^**−3**^**)**	**Material type**	**Electron affinity (eV)**
ZnO	100	3.3	1 × 10^16^	n type	3.87
WS_2_	200	1.3	6 × 10^21^	n type	4.3

## 2 Experimental design, materials, and methods

### 2.1 Sample preparation

The FTO glass was used as the substrate for the ITO/WS_2_/ZnO/FTO fabrication and was cleaned prior to the fabrication process described in Ref. [1].

The ZnO layer was fabricated according to conditions presented in Ref. [2].

The condition for WS_2_ layer deposition is presented as follow

**Table:** 

Target	WS_2_ (∅2 in., purity 99.999%)
RF power	50 W
Gas/Flow rate	Ar, 20 sccm
Working pressure	4 mTorr
Temperature	400 °C
Deposition time	10 min, 20 min, 30 min

The condition for ITO layer deposition is presented as follow

**Table:** 

Target	ITO (∅4 in., purity 99.999%)
RF power	300 W
Gas/Flow rate	Ar 50 sccm/O_2_ 0.3 sccm
Working pressure	5 mTorr
Temperature	Ambient temperature
Deposition time	10 min

### 2.2 Sample characterization

The quantities of various tungsten oxidation states are described in Fig. 1 by performing chemical analysis of the WS_2_ platelets on the Si substrate using X-ray photoelectron spectroscopy (XPS, PHI 5000 VersaProbe-II, ULVAC). Property of vertical WS_2_ layer was examining by transmission electron microscope (TEM, TALOS F200X) as presented by Fig. 2a. The chemical elemental mapping of WS_2_ platelets are presented by Fig. 2b with the detail distribution of W and S elements characterized by Fig. 2c using energy dispersive spectroscopy (EDS, JEOL, JSM_7001F). The diode properties of WS_2_/ZnO structure (rectifying ratio, ideal factor and barrier height) are presented by Fig. 3. The current-voltage property of WS_2_/ZnO device as presented by Fig. 3a was analyzed by using potentionstat/galvanostat (PGStat, ZIVE SP1, WonA Tech) using linear sweep voltammetry. The barrier height of 0.06 V of WS_2_/ZnO heterostructure is obtained by analyzing the flat-band potential of WS_2_/ZnO heterostructure which determined from the Mott-Schottky characteristics as presented by Fig. 3b. Fig. 4 presents the current-time property of the device at bias of ± 1. The illustration of band diagram of WS_2_/ZnO heterostructure which characterized by one-dimensional drift–diffusion equation solver program (SCAPS) [1] is based on material parameters presented by Table 1. The effects of RTP treatment and WS_2_ deposition time on ITO/WS_2_/ZnO/FTO device performances were studied by analyzing current-time characteristics of the device using chronoamperometry. The frequency and power of the light source were modulated by using a function generator (MFG-3013A, MCH instruments). Fig. 5 presents the photocurrent of the device under different temperature conditions of RTP process. And the correlation between photocurrent of the device and WS_2_ time deposition is presented by Fig. 6.
