# Supplementary material for: Physical and chemical data of WS2 platelets and thickness-dependent photoresponses
Source: Data Brief. 2018 Aug 30;20:1256–62. doi: 10.1016/j.dib.2018.08.118 (PMC6143772; doi:10.1016/j.dib.2018.08.118)
Supplement: Supplementary file 1 — Supplementary material [file mmc1.docx]

***Conflicts of Interest Statement***

Re: DIB-D-18-01868R1

Title: Physical and Chemical data of WS_2_ platelets and thickness-dependent photoresponses

We declare that this manuscript is original, has not been reported before, and is not currently being considered elsewhere. We also confirm that there is no known conflict of interest regarding this manuscript and its publication. The manuscript has been approved by all named authors.

Sincerely yours,


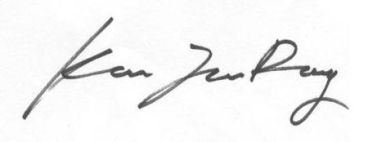


Joondong Kim

Joondong Kim, Ph.D./Professor

Department of Electrical Engineering,

Incheon National University

E-mail: joonkim@ incheon.ac.kr

Phone: +82-32-835-8770; fax: +82-32-835-0773
